# Tailoring Human Milk Oligosaccharides to Prevent Necrotising Enterocolitis Among Preterm Infants

**DOI:** 10.3389/fnut.2021.702888

**Published:** 2021-07-29

**Authors:** Safiyyah Abbas, Amy K. Keir, Maria Makrides, Laura D. Klein, Luke E. Grzeskowiak, Andrew J. McPhee, Alice R. Rumbold

**Affiliations:** ^1^Women's and Children's Health Network, Adelaide, SA, Australia; ^2^SAHMRI Women and Kids, South Australian Health and Medical Research Institute, Adelaide, SA, Australia; ^3^Adelaide Medical School, The University of Adelaide, Adelaide, SA, Australia; ^4^Robinson Research Institute, The University of Adelaide, Adelaide, SA, Australia; ^5^Business Growth and Innovation, Australian Red Cross Lifeblood, Sydney, NSW, Australia; ^6^College of Medicine and Public Health, Flinders University, Adelaide, SA, Australia

**Keywords:** preterm (birth), neonatal nutrition, oligosaccharides, breast milk, very low birth weight infants, necrotising enterocolitis

## Abstract

Necrotising enterocolitis (NEC) is a devastating disease affecting preterm infants, with little improvement in mortality rates and treatment strategies in the last 30 years. Human milk oligosaccharides (HMOs) are emerging as a potential preventive therapy, with multiple protective functions postulated. Our aim is to summarise the evidence concerning the role of HMOs in NEC development and emerging strategies to tailor the delivery of HMOs to preterm infants. Most research efforts to date have focused on supplementing preterm infants with simple oligosaccharides, which are structurally different to HMOs and derived mainly from plants. Clinical trials demonstrate limited benefits for NEC prevention arising from the use of these supplements. Alternative strategies under investigation include optimising HMOs for infants receiving donor human milk, concentrating oligosaccharides from donor human milk and from animal milks, as well as more sophisticated synthetic oligosaccharide production strategies. Critically, high quality evidence to support implementation of any of these approaches in the neonatal unit is lacking. Whether it is a specific HMO alone or a combination of HMOs that exert protective effects remains to be elucidated. Further challenges include how best to manufacture and administer oligosaccharides whilst retaining bioactivity and safety, including evaluation of the long-term effects of altering the balance of HMOs and gut microbiota in preterm infants. While several human clinical trials are underway, further research is needed to understand whether a tailored approach to oligosaccharide supplementation is beneficial for preterm infants.

## Introduction

Human milk oligosaccharides (HMOs) are complex carbohydrates that comprise the third most abundant solid component of human milk (1–5). Synthesised in the mammary gland, the basic HMO structure consists of a single glucose molecule at the reducing end attached to galactose, forming a lactose core (2, 6). Variation results from additional N-acetylglucosamine and galactose residues, and fucose or sialic acid at the non-reducing end (7–9). Over 200 structurally unique HMOs have been identified (2, 5, 10) and range in size from 3 to 32 sugars (6).

The three major classes of HMOs are fucosylated neutral HMOs, non-fucosylated neutral HMOs and sialylated acidic HMOs (11). Breast milk contains 80% neutral HMOs (12). Of these, 2′-fucosyllactose (2′-FL) (fucosylated) and Lacto-N-neotetraose (LNnT) (non-fucosylated) are the most abundant structures (13).

The amount and diversity of HMOs vary significantly based on the mother's stage of lactation, an infant's gestation at birth, and maternal genetic factors (1). HMOs are maximally concentrated in colostrum compared with mature milk (2, 14, 15). The ratio of fucosylated α1-2-linked HMOs to non-fucosylated HMOs changes from 5:1 to 1:1 over the first year of lactation (2). Variation in HMO composition is postulated to have crucial functional implications (9).

Milk from mothers delivering preterm contains a significantly greater concentration of HMOs than more mature milk, but there is great variation in concentration between mothers (6, 8, 14) and the HMO content is also less diverse (16). For example, compared with milk from mothers delivering at term, lacto-N-tetraose is generally more abundant (9), fucosylation is not as well-regulated (7, 9), and sialic acid content in the initial month postpartum is greater (7). However, these findings are not consistent across all studies of preterm and term milk (17).

Maternal “secretor” status and Lewis blood group are strong genetic determinants of HMO production, particularly concerning fucosylated HMOs, imparting four patterns of HMO fucosylation (1, 2, 5, 6, 18). Over 70% of women are “secretors,” meaning they express the α1-2fucosyltranferase *FUT2* gene, and produce α1-2fucosylated HMOs, such as 2'-fucosyllactose (2'FL) or lacto-N-fucopentaose 1 (LNFP1). “Non-secretor” women, who have homozygous mutations in *FUT2* and comprise 20% of Caucasian populations, do not produce these HMOs (2, 5, 13, 14, 19).

Lewis positive blood group women express α1-3/4-fucosyltransferase FUT3, producing α1-4-fucosylated HMOs such as LNFP2 (14). Combining Lewis blood group and secretor status has important implications. For example, Lewis blood group (a+b–) “non-secretor” milk contains 35–45% less total HMO amount than Lewis (a–b+) “secretor” milk (17).

## Role and Functions of HMOs

Due to the unique design of HMOs, their role in infant health and development has been under intense investigation during the past decade (8). HMOs are non-digestible sugars that are nutritionally beneficial not for the infant but for the bacteria residing in their gut (10, 20). A small proportion of HMOs are absorbed into the systemic circulation (5) and excreted through the urinary tract (10). The majority of HMOs that reach the gut are either passed in the stool or fermented in the intestine to short-chain fatty acids (SCFA) and lactic acids, creating an acidic environment unfavourable for many pathogenic microbes (2, 12, 18). HMOs selectively enhance the growth of beneficial bacteria, resulting in a healthy gut microbiome (21). Of the various gut microbiota species, only bifidobacteria and bacteroides can digest HMOs (21). Certain *Bifidobacterium* and *Bacteroides* species have been shown to efficiently utilise HMOs due to genes encoding specific enzymes involved with cleavage and transport of HMOs, in marked contrast other bacterial species (22, 23). *In-vitro* studies indicate LNnT, LNT, LNFP and 2'FL are preferentially digested by *Bifidobacterium longum* subsp. *infantis (B. infantis)*, and, to a lesser extent, *Bifidobacterium bifidum*. In contrast, pathogenic bacterial species show variation in HMO consumption. *Bacteroides fragilis* and *vulgatus* consume HMOs, but *Lactobacillus acidophilus, Clostridium perfringens, E. coli, Eubacterium rectale, Streptococcus thermophiles, E. faecalis*, and *Veillonella parvula* show limited or no utilisation of HMOs (8). Thus, the presence of HMOs, particularly fucosylated and sialylated HMOs, is thought to reduce the nutrients available for pathogenic bacteria, thus reducing their growth (19, 24, 25). “Secretor” milk may promote bifidobacteria species and protect against infant diarrhoea (13).

In addition to shaping the intestinal microbiota, evidence suggests additional antimicrobial and immunomodulatory roles of HMOs.

First, HMOs act as decoy receptors, competitively binding to pathogens by mimicking structurally homologous intestinal epithelial receptors, thus preventing pathogen attachment and invasion of the intestinal epithelium (2, 6, 8, 10). Sialylated HMOs, such as 3'SL, inhibit the binding of enteropathogenic *E. coli* (EPEC) (2, 18) in intestinal epithelial cells and uropathogenic *E. coli* (UPEC) in bladder epithelial cells (24). α1-2-fucosylated HMOs, such as 2'FL or LNFP1, inhibit the attachment of *C. jejuni* to the intestinal epithelium (14).

Second, HMOs may directly modulate immune cell responses to pathogens (14) and act as signalling molecules (24). For example, 2'FL directly inhibits lipopolysaccharide-mediated inflammation during *E. coli* invasion of intestinal epithelial cells (24). Other possible roles include leukocyte-endothelial cell and platelet-neutrophil interactions (2, 10), effects at the level of gut-associated lymphoid tissue (2), and interaction with selectins, integrins, and toll-like receptors (2, 26).

Third, HMOs affect intestinal epithelial cells, such as triggering intracellular processes such as differentiation and apoptosis, and reducing intestinal permeability in preterm infants during the first month of life (2, 14). In addition, HMOs may have other extraintestinal, indirect innate immune system effects, protecting against late-onset sepsis and urinary tract infections (6). Various other possible roles for HMOs have been postulated, including anti-allergic effects (2), and a role in growth and metabolism in the liver, muscle, and brain (2, 7, 10, 14, 19).

Research is needed to clarify the specific roles of HMOs and elucidate whether effects are due to one specific HMO, or a combination of HMOs interacting together (5). While speculative, it is highly likely that the beneficial effects of HMOs are dependent on several factors including total HMO amount, structural characteristics of individual HMOs, and abundance of specific HMOs (14).

HMOs are significantly more concentrated and structurally complex than milk oligosaccharides of any other species, including cow, sheep, goat, and non-human primates (2, 5, 6, 27). HMOs show greater complexity and diversity than non-human primate oligosaccharides, which are in turn more diverse than non-primates (28). Dairy animal milks contain more sialylated oligosaccharides, whereas fucosylated oligosaccharides predominate in human milk (4, 6, 10).

## Importance of HMOs for Preterm Infant Health and Development

Prematurity is associated with a higher risk of mortality and significant morbidities in infancy. HMOs may have a critical role in promoting a healthy gut microbiome and preventing bowel diseases such as necrotising enterocolitis (NEC).

NEC is a potentially life-threatening disease that affects 2–10% of very low birth weight (VLBW, i.e., <1,500 g) infants (7, 8). Characterised by intestinal inflammation, NEC can lead to bowel necrosis and perforation requiring surgery, with chronic complications including short gut syndrome, malabsorption, and neurodevelopmental delay (8, 29). Little has changed in NEC mortality rates—which can be as high as 25% in severe cases (8)—and treatment strategies over the last three decades (30).

The main risk factors for NEC are prematurity, pathogenic bacterial colonisation, and formula feeding (30). The premature gut is immature in several ways, with gut epithelium predisposed to mounting an exaggerated inflammatory response to pathogenic bacteria, resulting in the mucosal damage and impaired mesenteric perfusion implicated in the pathogenesis of NEC (8, 30). Increased toll-like receptor 4 (TLR4) signalling plays a role in this exaggerated inflammatory response (30–32). A bloom of pathogenic organisms, such as γ*-Proteobacteria*, is seen just before NEC onset (24, 25).

The most successful preventive strategies for NEC include feeds containing maternal milk and/or donor human milk, and probiotics (20). Preterm infants receiving their mother's milk are at six to ten times reduced risk of developing NEC than their formula-fed counterparts (3, 7). Suggested mechanisms behind the protective effect of breast milk against NEC include inhibition of TLR4 signalling (30), reduction of intestinal permeability (33), and promotion of a healthy gut microbiome through HMOs' selective enhancement of healthy bacteria (6, 10, 21).

The composition of the infant gut microbiome is strongly implicated in the development of NEC. Development of the gut microbiome occurs mainly due to breastfeeding (2). Various factors contribute to gut dysbiosis, including mode of delivery, antibiotic use, acid suppression, degree of prematurity, intestinal immaturity, lack of fresh breast milk, delayed introduction of enteral feeds, maternal gut microbiome composition, post-birth environment, and prolonged hospital stay with greater exposure to opportunistic infections (4, 5, 20, 25, 34).

The gut microbiota profile shows wide variability from the day after birth. Breastfed infants mainly show significant individual variation, and gut microbiota composition increases in amount and diversity with age (4). Compared with term infants, the preterm gut microbiota has low bacterial diversity, more potentially pathogenic gut flora strains, lower levels of *Bifidobacterium* and *Bacteroides*, immature digestive processes, and an immature mucosal barrier vulnerable to bacterial invasion and toxin damage (5, 8, 19, 34). All of these factors have been implicated in the development of NEC and sepsis (19, 24).

Animal studies suggest that disialyllacto-N-tetraose (DSLNT) (14) and 2'-fucosyllactose (2'FL) may be protective against NEC (35). Human observational studies support this with Autran et al. (3) and Van Niekerk et al. (1) finding breast milk low in DSLNT concentration was associated with an increased risk of NEC in the recipient preterm infants. Masi et al. (36) similarly found that DSLNT was significantly lower in maternal milk received by infants with NEC than age-matched controls. Infants who received milk with low DSLNT had lower relative abundance of *Bifidobacterium* spp. Further, Wejryd et al. reported lower HMO diversity and Lacto-N-difucohexaose I (which is only produced by secretor and Lewis positive mothers) levels in mothers of NEC cases, compared with non-NEC infants (37). There is evidence that preterm infants of “non-secretor” mothers show higher levels of *Proteobacteria*, which includes pathogens associated with NEC and sepsis (24). However, Demmert et al. demonstrated no differences in late onset sepsis or NEC in VLBW infants based on *FUT2* genotype (38).

This emerging evidence linking the HMO profile of maternal breast milk with risk of NEC has led to intense interest in understanding ways to improve the delivery of HMOs to vulnerable infants as a preventive strategy against NEC. Altering the profile of HMOs in maternal breast milk is difficult to achieve, as it is primarily determined by genetic factors and gestation at birth. Nevertheless, there are several possibilities regarding the use of donor human milk.

## Optimising HMOs for Infants Receiving Donor Human Milk

Donor milk is the preferred source of nutrition when sufficient maternal milk is not available (39, 40). While donor milk is associated with better health outcomes for preterm infants than infant formula, outcomes for donor milk-fed infants are not equivalent to those receiving maternal breast milk (41). This may be due to losses in milk nutrients or bioactives during storage and processing, or a mismatch in milk composition due to differences between donor and maternal lactation stage and/or maturity of the mammary gland (42). However, HMOs are one of few human milk components whose content and composition is unaltered by Holder pasteurisation (43).

Donor milk is typically prioritised for very preterm infants and used in the first weeks of life, when recipients would usually receive maternal colostrum or transitional milk. Yet donor milk is often mature milk from mothers who have given birth at term (29). The proportion of preterm donations to milk banks can vary considerably. For example, 65% of donors to an Indian milk bank had preterm births (44), compared with 10% of Taiwanese donors (45). This has important implications for preterm infants receiving donor milk, containing fewer HMOs than typical of breast milk from mothers who give birth preterm (14).

Donor and recipient matching may improve the nutrition and bioactives that infants receive. In a small observational study, Sánchez Luna et al. (46) observed a decrease in NEC rates in very preterm infants and late onset sepsis after implementing a personalised nutrition program that matches donor milk to recipient infants by gestational age and stage of lactation. While promising, donor milk matching programs require availability of milk from preterm donors, which may require targeted recruitment to increase the proportion of preterm donors, and staff resources to support labelling and matching. Increasing preterm donors could also increase similarities between donors' and recipient mothers' HMO profiles. However, this cannot be assumed given the variability in HMO content from mothers who deliver preterm (6, 8, 14). Additional studies are needed to compare nutrition and bioactives from gestational and lactation stage-matched donor milk to standard donor milk and to robustly evaluate the health benefits of such programs.

Matching donor milk to maternal secretor status may be a potential strategy to personalise donor milk, and provide infants of non-secretor mothers with milk from a secretor donor. An ongoing clinical trial is evaluating the impact of this on the gut microbiome among very preterm infants (47). Alternatively, increasing the diversity of HMOs in donor milk may be beneficial, for example, to include fucosylated HMOs (19, 25) and HMOs found in secretor milk (48). Pooling milk from multiple donors is already recommended to reduce variation in the macronutrient content of donor milk (49). More data about the composition and concentrations of key HMOs for preventing complications such as NEC or improving growth will be required to inform optimal milk pooling practises.

## Developing Concentrated HMO Supplements From Donor Milk

The development of human milk-derived supplements for preterm infants has been an area of intensive research and commercial interest, resulting in the use of at least one commercially available human-milk derived fortifier in neonatal units in the US (50, 51), used predominantly to increase protein intake. While there has been limited independent evaluation of the HMO content of this product, its product description states that the HMO content is similar to fresh milk (52). Meta-analysis of the evidence from the two published trials evaluating this product indicates the risk of NEC is reduced when this product is used compared with a bovine milk-based fortifier product. However, the overall quality of evidence was rated as low (53).

To date, there has been one published randomised trial of a supplement designed explicitly to produce a concentrated HMO product from donated human milk. The trial by Underwood et al. (20) examined two strategies. The first involved comparing infants fed formula supplemented with increasing doses of either a synthetic oligosaccharide product or an experimental donor human milk product containing concentrated HMOs. The second involved comparing infants fed maternal milk fortified with a commercially available human milk fortifier or a bovine milk fortifier. None of the interventions resulted in significant increases in faecal bifidobacteria, and there was a trend towards increased γ-Proteobacteria in the two experimental groups. The study was limited by the small sample population (*n* = 27 in total) precluding examination of clinical outcomes, and the high use of antibiotics in one of the human milk groups. Thus, the evidence for supplements that pool human milk oligosaccharides remains limited and in need of further investigation. Ongoing studies of new human milk-derived products are underway (54, 55).

## Oligosaccharide Supplements Derived From Dairy Species

Concentrating non-human sources of oligosaccharides have been proposed as an alternative supplementation strategy for preterm infants, but species specificity and lower concentrations of oligosaccharides in dairy animal milks pose challenges for development (2, 5, 6). For example, humans only produce one sialic acid residue, N-acetyl neuraminic acid (Neu5Ac), whereas other species produce other sialic acids (10). Studies focused on developing bovine milk oligosaccharides (BMO) reveal low concentrations of sialic acid overall, which is thought to be important to neurodevelopment and immunity (2), as well as lower HMO complexity (18). Further, most HMOs are fucosylated, which is not observed in BMOs (6).

Nevertheless, in animal models, BMOs are well-tolerated. They have been associated with increased stool frequency, softer stools, and greater bifidobacteria numbers, but may have limited benefits for gastrointestinal infections, respiratory infections, allergic responses, and growth (56). Goat milk oligosaccharides are another option, with greater concentrations of oligosaccharides than bovine milk (2). At present, there are no published trials involving animal-derived oligosaccharides given to human preterm infants, and there is limited evidence from trials examining the effects of BMOs given to healthy term infants (57).

## Synthetic Oligosaccharide Products

Years of commercial interest have resulted in commercially available oligosaccharides produced from plants or lactose, many of which are now added to infant formula for term and preterm infants. Long-chain fructo-oligosaccharides (lcFOS) contain fructose and are derived from inulin. Neutral short-chain galacto-oligosaccharides (scGOS) include galactose polymers from different fungi, yeast, and bacteria. Commercially available preparations often include a mixture of 90% scGOS and 10% lcFOS, which is proposed to mimic the prebiotic effect of neutral HMOs (12). Another type is pectin-derived acidic oligosaccharides (pAOS) (2, 20, 34). Reported side effects are attributed mainly to scFOS and are mild, such as flatulence (12), but overall these synthetic oligosaccharides appear to be well-tolerated (58).

The most recent systematic review of human clinical trials of synthetic oligosaccharides supplements (largely scGOS and lcFOS) in preterm infants was published in 2019 by Chi et al. (34). Meta-analyses revealed supplementation was associated with significant decreases in the incidence of sepsis, mortality, length of hospital stay, and time to full enteral feeding; but no significant difference in the risk of NEC or feeding intolerance.

Trials of synthetic oligosaccharides may show a lack of effect on NEC because lcFOS and scGOS are structurally much simpler than HMOs, and lack the diversity and unique effects of HMOs, such as bacterial specificity (2, 20, 59). Further, the current body of evidence is limited by major variation in the way these oligosaccharides are delivered to preterm infants. For example, trials have been undertaken comparing the addition of supplements among exclusively formula fed infants, whereas others have been restricted to exclusively breastfed infants or infants fed a mixture of breast milk and formula. Thus, it has not been possible to reliably tease out the effects of the supplement *per se*, from the effects of increased breast milk intake or complete removal of formula from the diet. In addition, most studies have been small and may therefore lack statistical power to show important effects with regard to NEC. Ongoing studies evaluating oligosaccharide products in preterm infants are underway and may help to shed light on these issues (60).

Results of animal studies have generally been mixed. In neonatal rat studies, the HMO disialyllacto-N-tetraose (DSLNT) (61) and enzymatically sialylated GOS or 2'FL (62) demonstrated protection against NEC. Further, 2'-FL and/or 6'-SL reduced NEC in mice and piglet models, and inhibited TLR4 signalling in human intestinal cells *in vitro* (31). Preterm pigs showed no benefit from oligosaccharides in terms of clinical outcome, systemic immunity, or gut function, flora or health (56, 63), which may suggest that HMO effects are seen only when the gut attains a certain degree of development (29). In a mouse model, pooled HMOs decreased EPEC attachment to and invasion of epithelial cells, but scGOS did not (8).

Other data suggest that some synthetic oligosaccharides can successfully act as decoy receptors. *In vitro* studies show that synthetic 2'FL and 3FL decrease adhesion of *C. jejuni, Pseudomonas, EPEC*, and *Salmonella enterica* serovar Fyris to Caco-2-cells (8). Additionally, scGOS can mimic the protective effect HMOs have on intestinal epithelial cells against *Entamoeba histolytica* cytotoxicity (8).

More recently, there have been advances in producing synthetic oligosaccharide structures that are identical in structure to those in human milk. To date, less than ten of the many hundreds of HMOs have been able to be replicated using chemical processes and microbial production (64). While this represents an exciting advancement in the field, trials proving safety and efficacy in preterm infants have not yet been undertaken.

## Discussion

**Figure 1:**
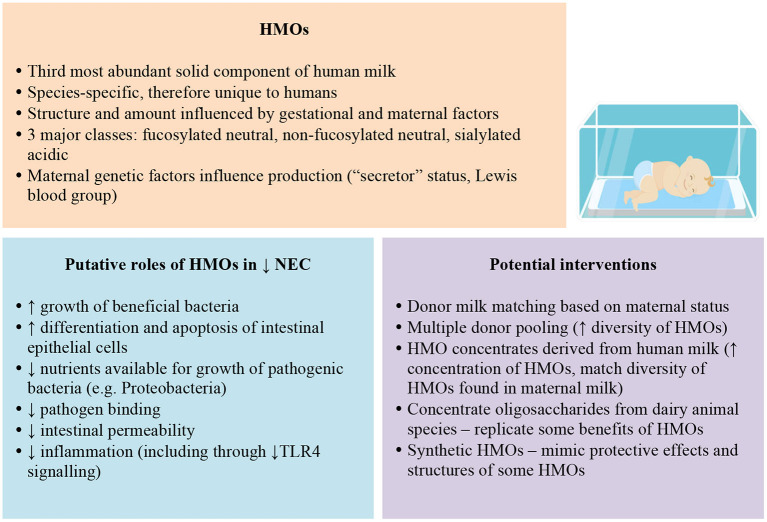
Potential HMO mechanisms and interventions to prevent NEC in preterm infants.

Foremost, strategies to support mothers of preterm infants to breastfeed must be paramount given the clear evidence of benefit of maternal breast milk for NEC prevention and infant growth and development. Beyond this, other methods to optimise intake of HMOs lack evidence of efficacy for NEC prevention and safety. Nevertheless, HMOs have plausible roles in protection against NEC and sepsis in preterm infants that warrant further investigation (Figure 1). For infants that lack access to maternal milk, altering the profile of donor milk, either via matching donor and recipients based on gestation or secretor status or by pooling donor milk, could increase HMO intake, but to date, all of these strategies lack rigorous evaluation.

Supplementation with synthetic oligosaccharides, predominantly scGOS and lcFOS, suggests benefits for some neonatal morbidities but trials have failed to demonstrate conclusive evidence that the risk of NEC is reduced. The relatively simple structure of scGOS and lcFOS suggest that they may be unlikely to mimic the complex functions of HMOs. The production of synthetic oligosaccharides identical in structure to HMOs also appears promising but all are yet to be tested in trials with preterm infants. Also lacking are studies of optimal dosing.

A critical limitation of research efforts to date is the focus on a limited number of oligosaccharide structures (7), thus negating the potential benefits of the wide variety of HMOs structures in existence. HMOs are produced as complex mixtures with possible synergistic mechanisms of action against NEC and infection (65). There is also a risk that supplementing feeds with just one or two of the many HMOs may cause an unwanted effect on the balance of the gut microbiome population and/or the immune system, with potential long-term implications.

Isolating HMOs from human milk would capture the diversity and complexity of HMOs and may be possible with the growth in human milk banking worldwide but presents other challenges. Products derived from donated breast milk are often processed to reduce risk for medically fragile infants. While low-temperature pasteurisation does not impact the amount or structure of HMOs, high-temperature sterilisation reduces HMOs (9, 66, 67). Heat treatment may also reduce the amount and activity of other milk components (e.g., B and T cells, soluble CD14, growth factors, vitamins, SIgA, lysozyme, Lactoferrin) (8, 9, 29) and it remains essential to ascertain whether the loss of the original human milk composition also results in functional changes to early microbial-HMO interactions (68).

Further, high quality research is needed that focuses on elucidating the mechanisms underlying the specific and synergistic effects of HMOs to inform the development of therapeutic applications. Any future HMO therapies require rigorous testing in trials that are of sufficient size to detect differences in NEC and other important clinical outcomes, to ensure there is robust evidence of efficacy and safety.

## Author Contributions

AR and SA conceptualised the paper and wrote the first draft, with input from AK and LK. All authors contributed to reviewing drafts and refining the paper and agree to be accountable for the content of the work.

## Conflict of Interest

LK works at Australian Red Cross Lifeblood, which supplies pasteurised donor human milk to hospitals in Australia. The remaining authors declare that the research was conducted in the absence of any commercial or financial relationships that could be construed as a potential conflict of interest.

## Publisher's Note

All claims expressed in this article are solely those of the authors and do not necessarily represent those of their affiliated organizations, or those of the publisher, the editors and the reviewers. Any product that may be evaluated in this article, or claim that may be made by its manufacturer, is not guaranteed or endorsed by the publisher.
